# The effect of brief digital mindfulness-based interventions on increasing online charitable behavior in Chinese adolescents

**DOI:** 10.3389/fpsyg.2023.1213089

**Published:** 2023-08-02

**Authors:** Mei Hong, Dapeng Liang, Teng Lu, Shengchen Liu

**Affiliations:** ^1^School of Management, Harbin Institute of Technology, Harbin, Heilongjiang, China; ^2^Harbin Institute of Technology, Harbin, Heilongjiang, China

**Keywords:** empathy, social connectedness, self-compassion, digital mindfulness based interventions, Chinese adolescents, donations

## Abstract

Digital mindfulness-based interventions (d-MBIs) have garnered significant research interest in recent years due to their psychological benefits. However, little is known about their impact on prosocial behaviors. This study investigates how d-MBIs impact prosocial behaviors where time spent is money, with Chinese adolescents as the subjects, through an online charity task (www.freerice.com). 119 students from a high school in China, who were inexperienced with mindfulness meditation, participated in this randomized controlled trial. The d-MBI group (*N* = 39) received online MBI guidance, while the face-to-face mindfulness-based intervention (f-MBI, *N* = 43) group underwent mindfulness intervention under personal tutors. The active control group (*N* = 37) completed a crossword task. Data analysis first involved repeated measures variance analysis, including pre-and post-intervention assessments. Subsequently, a two-way variance analysis was performed, with gender (female and male) and group (d-MBI, f-MBI, active control) as independent variables and the number of grains as dependent variables for the three groups of participants. Results showed that d-MBIs effectively improved empathy and compassion in Chinese adolescents, leading to increased rice donations to the United Nations World Food Program. These results underscore the positive effect of d-MBIs on prosociality and suggest their applicability in beneficial real-world situations involving prosocial behaviors, extending beyond previous research primarily conducted in artificial and hypothetical scenarios.

## 1. Introduction

Mental health issues impact between 10 and 20% of children and adolescents worldwide (Kieling et al., 2011). Adolescents, typically those born around 2008 and aged between 11 and 18, are considered digital natives in today’s internet-dominated world (Zhu et al., 2022). Recent research has found that using digital products has become an addiction, profoundly affecting adolescents’ social lives and well-being (Mason et al., 2022). In response to the mental health needs of this demographic, an increasing number of digital technologies, such as mobile applications and web delivery programs, are being developed and applied (Bergin et al., 2020). Consequently, the development and utilization of digital tools that promote socio-psychological health are experiencing explosive growth. Researchers are increasingly reporting these tool’s immense potential and value (Lucas-Thompson et al., 2019; Sommers-Spijkerman et al., 2021).

During adolescence, the social world of teenagers becomes increasingly essential. Peer networks expand, intimate friendships surpass parental support, and romantic relationships develop (La Greca and Harrison, 2005). Although this is a vulnerable period in adolescent development, it also represents a “window of opportunity” for fostering positive psychological growth (Graber and Brooks-Gunn, 1996). Positive interpersonal relationships and peer acceptance provide numerous psychological benefits, affecting self-esteem and protecting against social isolation (Hall-Lande et al., 2007). Adolescents with healthy peer relationships and high levels of peer support are less likely to engage in risky behaviors (Telzer et al., 2014). In contrast, peer rejection is associated with internalizing and externalizing problems such as social withdrawal, low self-esteem, depression, conduct disorder, and attention deficits (Cheang et al., 2019). These findings suggest that positive interpersonal relationships and peer acceptance positively impact the psychological well-being of children and adolescents, contributing to their overall development.

Prosocial behavior, a crucial aspect of peer acceptance, is defined as actions that are in the best interest of others rather than oneself, such as helping, comforting, sharing, cooperating, charitable giving, and volunteering (Eisenberg and Miller, 1987; Denham et al., 1990). Such behavior is linked with empathy and self-compassion in adolescence (Marshall et al., 2020). In this study, we narrow the definition of prosocial behavior to charitable giving, specifically focusing on donations made by Chinese adolescents to support online charitable causes. This focus represents a direct and widely practiced form of altruism.

One suggested method to increase prosocial behavior is to enhance mindfulness (Schindler and Friese, 2022). Mindfulness involves intentionally focusing on the present moment without judgment while maintaining a non-reactive and compassionate attitude (Kabat-Zinn, 2015). The potential role of mindfulness in educational settings has piqued the interest of scientists, educators, and policymakers, spurred by a growing body of evidence. Systematic reviews reveal that mindfulness-based interventions (MBIs) positively impact prosocial outcomes and promote helping behaviors, with medium effect sizes (Kreplin et al., 2018; Donald et al., 2019). Interestingly, short interventions of less than one hour and more extended interventions exceeding ten hours have similarly powerful effects. Consistent with these findings, a meta-analysis of 72 studies concluded that MBIs foster flexible emotion regulation, such as enhancing the capacity to modulate empathetic responses and default self-perspectives (Hoge et al., 2021). Thus, increased flexibility might facilitate prioritizing others’ welfare over personal preferences (Batson, 2010). Additionally, MBIs have been found to heighten self-compassion among adolescents, prompting changes in prosocial behaviors, bolstering peer acceptance, and improving mental health (Cheang et al., 2019). Overall, MBIs have shown considerable effectiveness in fostering prosociality, with empathy and self-compassion being crucial components (Cameron and Fredrickson, 2015).

Implementing mindfulness training programs for students involves providing face-to-face guidance through trained mindfulness instructors, a significant component of these initiatives (Meiklejohn et al., 2012). However, logistical and financial hurdles accompany the implementation of these programs (Semple et al., 2017). Firstly, training competent mindfulness instructors can be both resource-intensive and costly. These professionals require rigorous and extensive training to ensure they possess the knowledge and skills to teach mindfulness effectively. Schools must invest in these instructors’ continuous professional development to align them with the latest research, techniques, and best practices in the field. These compounded factors make it challenging for schools to ensure that the mindfulness training offered to students is evidence-based (Mrazek et al., 2019a,b).

The rapid development of digital mindfulness-based interventions (d-MBIs) in recent years has facilitated the delivery of high-quality training to an increasingly interconnected global population through the Internet. Mobile applications and web-based platforms potentially surpass traditional face-to-face modes, enhancing the accessibility, standardization, customization, and efficacy of mindfulness training. Preference for online mindfulness interventions over group sessions has significantly increased (Wahbeh et al., 2014), underscoring the substantial potential of digital media in advancing public health. A recent meta-analysis by Spijkerman et al. (2016) found substantial benefits of digital mindfulness training on factors such as stress, anxiety, depression, and overall well-being, suggesting a level of efficacy potentially comparable to in-person training. This viewpoint is further corroborated by other recent studies, which indicate that digital mindfulness training can enhance life quality (van Emmerik et al., 2018), improve well-being (Howells et al., 2016), increase self-reported mindfulness (Plaza García et al., 2017), and reduce symptoms of depression (Ly et al., 2014).

Although mindfulness correlates positively with prosocial behavior in the real world, it remains unclear whether this association extends to the online environment. A significant difference between digital mindfulness-based and traditional face-to-face training is that the former is self-directed, and thus relies on its effective design and implementation. However, many current mindfulness apps fall short in applying best practices in digital learning, leading to only moderate evaluations in terms of engagement, functionality, and information quality (Mani et al., 2015). These issues may be more pronounced in school settings, where students do not self-select into the interventions based on prior interest or motivation. For instance, in a study requiring students to complete a d-MBI, only one out of 85 students managed to complete the whole course (completion defined as doing at least 40 out of 96 exercises) (Antonson et al., 2018). Therefore, for d-MBIs to be successful, they must innovatively apply best practices in digital learning. This includes tailoring instructional content, utilizing sound pedagogical methods, addressing audience diversity, increasing student engagement, and predicting and addressing potential challenges (Mrazek et al., 2019a,b). Consequently, this study aims to investigate whether a d-MBI designed for adolescents can be effectively implemented in a school environment and positively impact their prosocial behavior. The importance of this research lies in verifying if the introduction of d-MBIs at the compulsory education stage would be a beneficial strategy to enhance adolescents’ prosociality, which could positively affect their ability to respond to the needs of others.

It is worth noting that previous research on the relationship between mindfulness and prosocial behavior has mainly used monetary donations as an indicator of charitable behavior, and the core of such research focuses on assumed or artificially created helping behavior scenarios. Although this is common, it is not the only form of charity, and such a narrow focus may limit the application to other forms of charity. For example, research has also used non-monetary prosocial tasks, such as daily prosocial behaviors (Lockwood et al., 2017), blood donation (Hong and Lee, 2021), voting (Matland and Murray, 2016), and volunteering time to charitable organizations (Macchia et al., 2023), to empirically test theories of prosocial behavior. In addition, previous research on mindfulness training mainly used inactive control groups (i.e., waitlist or no intervention) (Ly et al., 2014; van Emmerik et al., 2018). Therefore, this study applies digital mindfulness interventions to real-world scenarios, such as actual charity activities where it is not money that is requested. It investigates the effect of d-MBIs on adolescents’ prosocial behavior through the innovative application of a real-world online charity organization. This will be compared with the level of altruistic behavior observed in the case of f-MBIs or active control.

Online prosocial behavior is measured via a unique real-world online charitable task,^1^ where the contribution is time spent, not money. This is a particularly relevant behavioral change because one direct interpretation of MBIs leading to prosocial behavior is that MBIs can induce feelings of compassion and empathy in teenagers, thus motivating and increasing subsequent prosocial behavior (Cheang et al., 2019). Especially when charitable donations occur in single, repetitive actions (e.g., dropping coins into a donation box one by one), this emotional shift can occur instantly. However, by investigating time spent as a charitable act, this study will be able to assess how MBIs contribute to online charitable behavior over a longer period. During this time, positive emotions such as empathy and compassion may have a more lasting impact on prosocial decision-making than the MBIs themselves.

The primary research question of the current study was designed to ascertain whether d-MBIs tailored specifically for high school students would be viable in a school environment. The secondary research question was to determine if the effects of d-MBIs on adolescents’ prosocial behavior were comparable to those of traditional f-MBIs. Drawing from previous research, we formulated the following hypotheses:

*H1*: Participants in the intervention group will dedicate more time to the charitable task than participants in the active control group.

*H2*: d-MBIs and f-MBIs will similarly on participants’ online charitable donation behavior.

## 2. Methods

Ethical approval for this study was provided by the Ethics Committee of Harbin Institute of Technology. Following Grepmair et al. (2007), a randomized, double-blind, active-controlled design was used to evaluate the impact of f-MBIs, d-MBIs, and active control conditions on online charitable donation behavior. Participants were blinded to the group and research hypotheses. Similarly, experimenters were unaware of the treatment allocations (detailed below).

### 2.1. Participants

The study sample consisted of 119 teenagers from a vocational senior high school in Yantai, Shandong Province. They were randomly assigned to the d-MBIs (*n* = 39), f-MBIs (*n* = 43), and active control (*n* = 37) groups.

Participants were recruited between September and November 2022. The recruitment was done by promoting in different classes of 10th to 11th grades at the high school. The target population was typical teenagers (average age of 16.69 ± 1.01, range 15–18). This sample complements the study’s design as all the participants were first-time meditation. Furthermore, participation in this study has potential benefits for the target population, as previous reports suggested that meditation could reduce stress and anxiety related to students (Lemay et al., 2019), and improve academic performance (Lin and Mai, 2018). Informed consent for all participants in the study was obtained through the popular Chinese professional survey website *Wenjuanxing*^2^ (a website like SurveyMonkey). Participants could only proceed with the study upon agreement with the informed consent.

We conducted the survey in the following steps. First, we signed informed consent forms with the principal of the school and the homeroom teachers of each selected class. Then, once the students agreed to participate, our experimenters obtained the parents’ contact information from the school and used this information to gain consent from the children and their parents to participate in the research. We informed the parents or guardians of the students that if they did not want their child to participate in the survey a week before the screening day, they should contact the teacher by phone. Next, we explained to the students the anonymity and confidentiality of the data, but not the subject and purpose of the research. They could opt out of the research if they did not want to participate. As the data was anonymous and the research did not include risks or violations to the participating students’ health and rights, this study was conducted by the Helsinki Declaration.

Exclusion criteria for the sample were having previous meditation experience or having participated in some mindfulness training. This procedure was carried out to balance the participants’ experience level, a potential confounding variable. In addition, we also checked if there were students who exhibited a high level of altruism and excluded him/her from the study; we confirmed that no student reported a significant level of empathy and willingness to help ( p>0.05 ). All students participated in this study voluntarily.

The power analysis was based on a meta-analysis of the relationship between MBIs and prosocial behavior (Luberto et al., 2018). The sample size was determined using G*Power, estimating that 90 participants were required. The anticipated effect size was f=0.25 ( α=0.05 , statistical power =0.90 ).

### 2.2. Procedures

This web-based survey was held in the Student Activity Center for 3 days, from 8 a.m. to 6 p.m. Upon arrival, the participants were randomly seated in computer booths, that ensured anonymity. All experimental procedures were conducted on iPads. After reading and signing the digital informed consent form, participants answered questions regarding sociodemographics (gender, age, ethnicity, academic year) and their eligibility for the study. They were only allowed to participate in the study when they indicated that they were currently attending high school (target audience) and had no previous experience in studying Buddhism or meditation (exclusion criteria). Before randomization, participants completed questionnaires that included social connectedness, self-reported empathy, and self-compassion measures. Subsequently, participants were randomly assigned to the d-MBI, f-MBI, or active control group. Randomization was automatically carried out via *Wenjuanxing* based on the record number of participants (student ID) obtained at the start of the study. As a result of the randomization, each respondent was automatically directed to the treatment corresponding to their condition. The three groups showed no significant differences in gender, age, or grade, and sociodemographic variables were evaluated.

Before the formal start of the experiment, participants in the intervention group (d-MBI and f-MBI group) took a mindfulness course, which explained some aspects of the basic instructions (the content of awareness), as well as the emotions that come with practice (such as non-judgment, acceptance, gratitude, and generosity). The aim of these meditations was to direct attention to breathing and bodily sensations, while trying not to identify with the mental events that occur during this process.

During the formal experiment, the interventions for the d-MBI and f-MBI groups were different: participants in the d-MBI group received a pre-recorded digital material with mindfulness intervention, while the f-MBI group underwent interventions applied by two tutors with extensive experience in mindfulness and compassion training management. The intervention content, target, and the voice of the tutors were identical for both groups. Participants were asked to engage in the entire course earnestly. Meanwhile, participants in the active control group watched a 40-min video on ancient poetry appreciation and then completed a poetry crossword game. This cognitive activity implied an elevation in the participants’ attention level during the experiment (Brooker et al., 2019), comparable to the level of attention required for mindfulness (Norris et al., 2018). Furthermore, this task paralleled the conditions of the intervention groups, avoiding group factors and differences in social interaction that could influence altruistic levels.

Within 10 min of the end of the charity task, participants immediately undertook a second self-report evaluation. We informed participants that their effort would contribute to charitable causes, but there was no further reward. Participants began answering questions on www.freerice.com on the tablet device and were informed they could play the game as they wished. After the study, students in the control group were invited to attend a mindfulness course similar to those in the intervention group. All participants received a charitable souvenir. Thus, the experiment involved no deception and was conducted in an incentive-compatible manner, adhering to the standards of economic research (Schram, 2005).

#### 2.2.1. Mindfulness interventions

In the intervention groups, interventions were based on standardized mindfulness and compassion training to promote altruistic behavior by developing compassion for others and enhancing moral awareness. The students learned to (1) focus attention and stabilize their state of mind; (2) regulate emotions and cultivate self-compassion; (3) cultivate compassion for others; (4) enhance moral awareness. The intervention drew inspiration from the short-term mindfulness meditation training audios developed by Wu et al. (2019) (JW2016 Version) and the brief mindfulness intervention based on mindfulness and compassion (González-García et al., 2021).

The intervention content for both groups was identical. However, a specific online platform was created for the d-MBI group. In contrast, we hired two psychologists for the f-MBI group, with over ten years of experience delivering MBIs to adolescents and adults. The intervention was comprised of three modules on different themes (total intervention time: 60 min), each module containing: (1) a mini-lecture (10 min), and (2) formal mindfulness and compassion practices (guided meditation) (10 min). The detailed outline of the program can be seen in Table 1. These interventions involved the basic aspects of mindfulness and compassion and applying the mindful attitude in prosocial decision-making across different experience contents (sensations, thoughts, and emotions).

**Table 1:** Outline of the d-MBIs: summary of modules, components, and topics covered.

Modules and educational objectives	Component and topics covered
Module one: enhancing mental health and awareness	
Minilecture: “Main Difficulties in Mindfulness and How to Handle Them” (10 min)	A deep dive into the challenges of mindful practice, offering strategies for overcoming them and dispelling common myths and misconceptions. The session emphasizes the right intentions, ethical awareness, mindfulness attitudes, and the role of physical awareness and embodiment.
Body Scan meditation (10 min)	This session guides participants on a journey of mental equilibrium through the Body Scan meditation technique. It involves directing and maintaining attention to physical sensations throughout the body, and encourages the cultivation of mindfulness attitudes. This practice fosters greater bodily awareness and can lead to improved focus, relaxation, and overall well-being.
Self-Reflection and Rest (5 min)	
Module two: promoting emotional regulation through self-compassion	
Minilecture: “Mindfulness and Self-Compassion” (10 min)	An exploration of the relationship between mindfulness and self-compassion, with insights on how to cultivate self-compassion through mindfulness for improved mental well-being and resilience.
Self-Compassion Meditation (10 min)	This session guides participants through a transformative journey of developing the three crucial elements of self-compassion: mindfulness, common humanity, and kindness. Especially designed for challenging moments, this meditation practice aims to nurture a sense of understanding, acceptance, and love toward oneself, promoting personal growth and resilience.
Self-Reflection and Rest (5 min)	
Module three: boosting resilience through moral responsibility and compassion	
Minilecture: “What is Compassion and how it can help you?” (10 min)	A comprehensive exploration of compassion, its distinction from empathy, its benefits, and how it can enhance mental and physical health, reduce stress, and improve relationships. The session also introduces practical ways to cultivate compassion.
Compassion in Action Meditation (10 min)	This session guides participants to foster awareness, compassion, and a heightened sense of moral responsibility in their interactions with others. By mindfully integrating these principles in daily life, participants learn to navigate social situations with greater empathy, understanding, and ethical consciousness.
Self-Reflection and Rest (5 min)	

To overcome the challenges that d-MBIs might bring, we applied some of the best digital learning practices suggested by Mrazek et al. (2019a,b) and González-García et al. (2021). First, we targeted adolescents aged 11–18, a population that our research group understands well, so we had accurate information about the challenges they face, allowing us to select the most relevant training outcomes for them. Secondly, to promote engagement and effective learning, we constructed the three modules of the intervention as short information fragments that were interesting and appealing to our target audience. The mini-lectures consisted of videos featuring mindfulness instructors, displayed from the waist up, on one side of the screen, elucidating the primary topics. On the other side were slides filled with engaging content. The audio instructions included a one-minute guided preparation and a 10-min mindfulness intervention that integrated the cognitive notions of mindfulness and meditation training techniques, adopting a breath observation meditation method that is time-efficient, economical, and easy to promote. This has significant application value for groups who lack teachers, time, and money but could benefit from mindfulness meditation.

#### 2.2.2. Online charitable task

Freerice (see text footnote 1) is a website supporting the United Nations World Food Program (WFP). Previous research used the site as a pro-social task for non-monetary donations (Farrelly and Bennett, 2018). On this platform, individuals can answer endless multiple-choice questions about various topics. For every correct answer provided, Freerice donates ten grains of rice to the WFP. The amount of rice donated by the participants is displayed on the screen when they answer the questions correctly, with a bowl of rice providing a visual representation. Therefore, the more rice grains in the bowl, the longer the participant has spent on the platform, effectively donating their time. Considering the English proficiency level of Chinese high school students in this experiment, we adopted a mode of translating Chinese vocabulary into English vocabulary and chose a simple difficulty level. Participants were required to find the correct definition of a word from four options (for example, “日” is: bright, moon, sun, follow).

### 2.3. Measures

The Social Connectedness Scale (SOC; Lee and Robbins, 1995) consists of 8 items designed to evaluate interpersonal connectedness and feelings of belonging. Participants indicate their responses using a 6-point Likert scale ranging from 1 (agree) to 6 (disagree). The SOC has been successfully applied to Chinese adolescents (Du and Wei, 2015), with a Cronbach’s alpha coefficient of α=0.95 (Mu et al., 2021). The possible scores range from 8 to 48 points, where higher scores signify a greater sense of connectedness. The reliability analysis for the SOC scale revealed that Cronbach’s α was 0.905 for scores before the intervention and 0.897 for scores after the intervention.

The Interpersonal Reactivity Index (IRI; Huang et al., 2012) is a self-report measure of 28 items, rated on a 5-point Likert scale. IRI subscales are grouped according to their cognitive or affective nature. On the cognitive side, perspective-taking and fantasy are assessed. On the affective side, empathic concern and personal distress. These subscales demonstrate good validity and variable reliability, with Cronbach’s alpha values between 0.56 and 0.70. Due to the study employing a real-life charitable task, mindfulness is associated with an increased focus on experiencing empathy and adopting others’ perspectives rather than on personal distress (Wallmark et al., 2013), so we retained only the perspective-taking and empathic concern subscales. The reliability analysis for these scales revealed that Cronbach’s α for pre-intervention scores was perspective-taking ( α=0.626 ) and empathic concern ( α=0.652 ), while for post-intervention scores, it was 0.631 and 0.661, respectively.

The Self-Compassion Scale-Short Form (SCS-SF; Raes et al., 2011) comprises 12 items. Participants rate their responses on a 5-point Likert scale. The SCS-SF has been extensively utilized in adolescent populations, including samples with an average age of 12–16 years (Ferrari et al., 2022)—the final scores for the SCS-SF range from 12 to 60 points. Higher scores reflect a stronger tendency toward kindness, common humanity, and mindfulness. Reliability analysis of the SCS-SF scale indicated that Cronbach’s α for pre-intervention scores stood at 0.885, while for post-intervention scores, it was 0.908.

## 3. Results

### 3.1. Attrition and baseline equivalence

**Figure 1:**
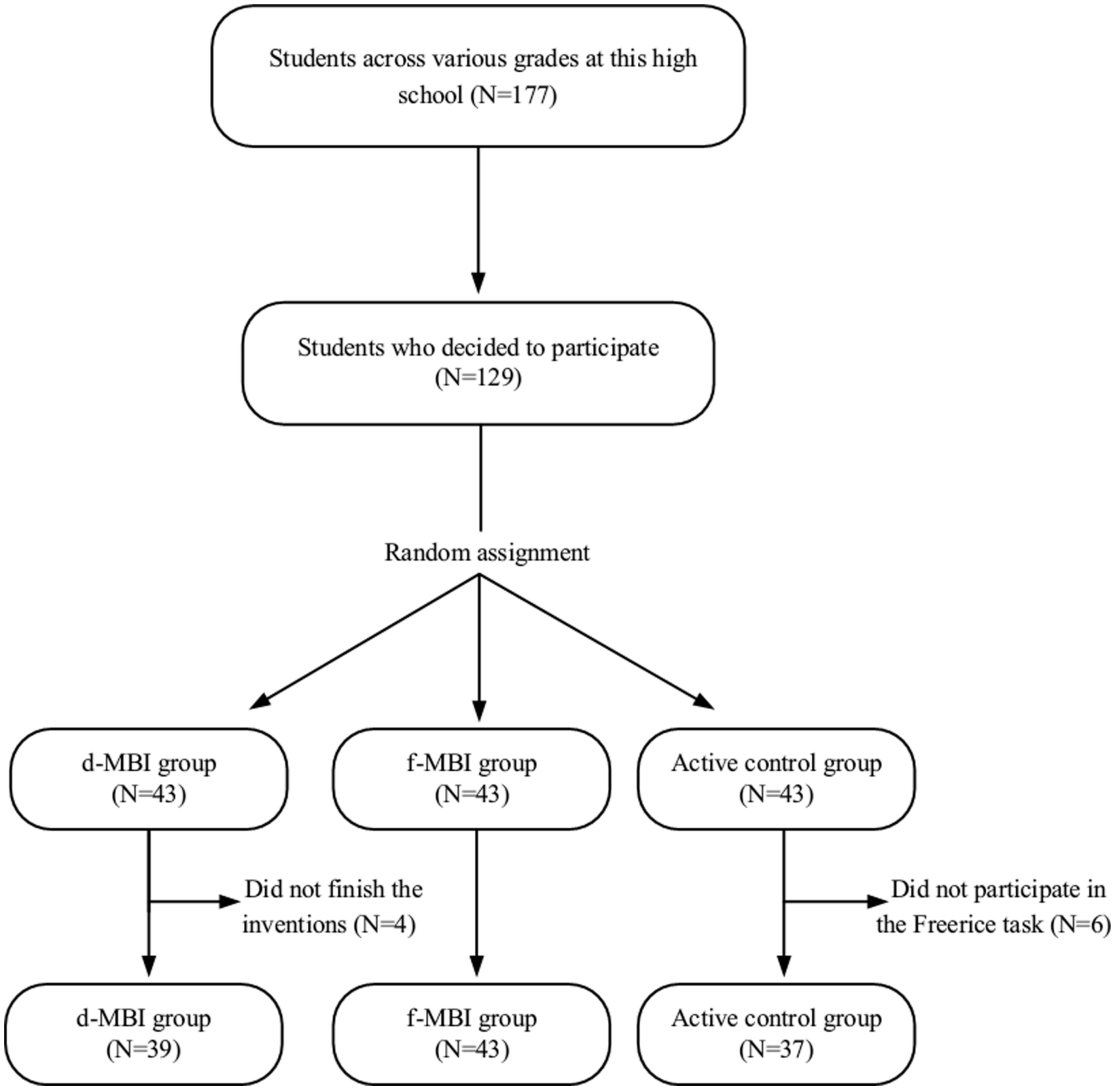
Assignment of the participants to the study groups.

Figure 1 shows the flow of participants in the study. A total of 177 people were initially recruited, of which 129 participants met the inclusion criteria and chose to participate in the experiment. Ten participants dropped out before completing the experiment, leaving a final sample size of 119 (62 females, 57 males). Among those who dropped out, 4 were from the d-MBI group, and 6 were from the control group, with attrition rates of 9.3 and 13.95%, respectively. The attrition rate for the control group was slightly higher, leaving final sample sizes of 39, 43, and 37 for the d-MBI, f-MBI, and control groups, respectively. The difference was due to the absence of an instructor in the d-MBI group, with some students dropping out because they were not studying seriously (a drawback of d-MBIs), while students in the control group reported feeling tired after completing the ancient poetry crossword puzzle and uninterested in the English vocabulary task in the Freerice task.

Descriptive statistics, Kruskal-Wallis H Test, and Chi-square tests were employed to outline and compare the baseline sociodemographic characteristics across groups (refer to Table 2). Continuous variables are presented as means with SDs, whereas categorical variables are depicted as counts and percentages. Results indicate no group differences in sociodemographic variables (all p>0.05 ) before intervention.

**Table 2:** Participant sociodemographic characteristics at baseline (*N* = 119).

	d-MBI group	f-MBI group	Control group	*p*
	N=39	N=43	N=37	
Gender				$\chi^{2}=1.84$
Male/female ( % male)	18/21(46.15)	18/25(41.86)	21/16(56.76)	p=0.399
Age				$\chi^{2}=3.54$
Mean (SD)	16.90(0.97)	16.49(1.01)	16.70(1.10)	p=0.170
Academic year				$\chi^{2}=0.17$
First year [yes/no ( % yes)]	26(66.67)	28(65.11)	23(62.16)	p=0.917
Household income				$\chi^{2}=0.96$
≥ CHY100000 [yes/no ( % yes)]	25(64.10)	23(53.49)	22(59.46)	p=0.619

Statistical comparisons between groups were made using the Kruskal-Wallis H Test for continuous variables and chi-square analyses for categorical variable.

### 3.2. Non-response bias

This study dealt with non-response bias by implementing the strategy proposed by Armstrong and Overton (1977), which includes applying Chi-square tests and the Mann–Whitney-Wilcoxon test for early and late respondents. All samples are sorted in the order of registration ID. The top 25% of respondents were considered earlier respondents, and the bottom 25% were considered later respondents. The results indicated no significant differences in all variables of demographics for these two stages ( p>0.05 ). Therefore, we excluded the possibility of non-response bias.

### 3.3. Common method bias

We performed two tests to address this study’s potential concern for common method bias. The first is Harman’s single-factor test (Podsakoff and Organ, 1986; Podsakoff et al., 2003). The results indicated four factors with initial eigenvalues greater than 1.00; the first factor accounted for 21.46% of the variance (not exceeding the threshold of 50%). Thus, the common method bias is not pervasive in this study. The second one used VIF as an indicator to test whether common method bias exists (Kock, 2015). In this study, all factor-level VIFs ranged from 1.019 to 2.078, lower than 3.3, so the model can be considered free of common method bias.

### 3.4. Effectiveness of the MBIs

Following verifying the assumptions of normality and homoscedasticity of the variables, which were assessed using the Shapiro–Wilk and Levene tests, respectively, we analyzed variance (ANOVA) to identify any significant differences among the groups concerning social connectedness, self-reported empathy, and self-compassion before the intervention. Concurrently, a repeated-measures ANOVA was carried out, incorporating pre-and post-intervention assessments. We examined the main effect of “time” (pre- and post-intervention) within different groups and the interaction effects (group × time). To estimate the effect size, we utilized partial eta squared ( $\eta_{p}^{2}$ ) with 90% confidence intervals (Steiger, 2004) for ANOVA analyses and Cohen’s d with 95% confidence intervals for pairwise comparisons, respectively.

#### 3.4.1. Social connection

In terms of social connectedness (Table 3), no significant differences were observed between groups prior to the intervention ( F(2,116)=0.08 , p=0.921 , $\eta_{p}^{2}<0.01$ , 90% CI [0.00,0.01] ). In the d-MBI group, a main effect of “time” on social connectedness was found, F(1,38)=4.00 , p=0.048 , $\eta_{p}^{2}=0.03$ , 90% CI [0.00,0.10] , with a significant increase between pre- and post-intervention values, t(1,38)=−2.00 , p=0.048 , Cohen’s d=−0.32 , 95% CI [−0.64,−0.00] . In the f-MBI group, a main effect of “time” on social connectedness was found, F(1,42)=5.95 , p=0.016 , $\eta_{p}^{2}=0.05$ , 90% CI [0.01,0.13] , with a significant increase between pre- and post-intervention values, t(1,42)=−2.44 , p=0.016 , Cohen’s d=−0.38 , 95% CI [−0.68,−0.07] . In the control group, F(1,36)=0.38 , p=0.539 , $\eta_{p}^{2}<0.01$ , 90% CI [0.00,0.04] , no significant differences were observed. The “group × time” interaction effect was not significant, F(2,116)=0.52 , p=0.597 , $\eta_{p}^{2}=0.01$ , 90% CI [0.00,0.04] .

**Table 3:** Social connectedness (SOC), self-reported empathy (IRI), and self-compassion (SCS-SF) in the different groups pre- and post-intervention evaluation, and intra-group effect sizes (*d*) between assessments.

	d-MBI group			f-MBI group			Control group		
	Pre	Post	*d*	Pre	Post	*d*	Pre	Post	*d*
	Mean (SD)	Mean (SD)		Mean (SD)	Mean (SD)		Mean (SD)	Mean (SD)	
Social connectedness									
SOC	32.92 ( 10.84 )	34.21 ( 10.20 )	−0.32	32.49 ( 10.24 )	33.98 ( 10.23 )	−0.38	33.43 ( 9.96 )	33.84 ( 9.59 )	−0.10
Self-reported empathy									
Perspective-taking									
PT	27.18 ( 5.35 )	27.67 ( 5.15 )	−0.33	26.61 ( 5.12 )	27.30 ( 4.94 )	−0.47	26.70 ( 5.48 )	26.92 ( 4.99 )	−0.14
Empathic concern									
EC	26.72 ( 5.17 )	27.33 ( 5.12 )	−0.57	26.37 ( 5.45 )	27.26 ( 5.37 )	−0.82	26.84 ( 5.20 )	27.08 ( 5.15 )	−0.23
Self-compassion									
SCS-SF	38.10 ( 12.85 )	39.18 ( 11.64 )	−0.33	37.63 ( 12.02 )	38.93 ( 12.61 )	−0.40	38.27 ( 11.16 )	38.62 ( 10.89 )	−0.11

#### 3.4.2. Self-reported empathy

Regarding self-reported empathy (Table 3), no significant differences were observed between the groups on the different subscales at pre-intervention assessment (perspective taking, F(2,116)=0.13 , p=0.875 , $\eta_{p}^{2}<0.01$ , 90% CI [0.00,0.02] ; empathy concern, F(2,116)=0.09 , p=0.918 , $\eta_{p}^{2}<0.01$ , 90% CI [0.00,0.01] ). For the d-MBI group, there was a significant main effect of “time” on the perspective-taking subscale, F(1,38)=4.05 , p=0.046 , $\eta_{p}^{2}=0.03$ , 90% CI [0.00,0.10] . A significant increase was observed when comparing pre- and post-intervention, t(1,38)=−2.01 , p=0.046 , Cohen’s d=−0.33 , 95% CI [−0.65,−0.01] . Similarly, a significant main effect of “time” was observed for the empathic concern subscale, F(1,38)=12.56 , p<0.001 , $\eta_{p}^{2}=0.10$ , 90% CI [0.03,0.19] . A significant increase was observed when comparing pre- and post-intervention, t(1,38)=−3.54 , p<0.001 , Cohen’s d=−0.57 , 95% CI [−0.89,−0.25] .

For the f-MBI group, there was a significant main effect of “time” on the perspective-taking subscale, F(1,42)=9.16 , p=0.003 , $\eta_{p}^{2}=0.07$ , 90% CI [0.02,0.16] . A significant increase was observed when comparing pre- and post-intervention, t(1,42)=−3.03 , p=0.003 , Cohen’s d=−0.47 , 95% CI [−0.77,−0.16] . Similarly, a significant main effect of “time” was observed for the perspective-taking subscale, F(1,42)=28.55 , p<0.001 , $\eta_{p}^{2}=0.20$ , 90% CI [0.10,0.30] . A significant increase was observed when comparing pre- and post-intervention, t(1,42)=−5.34 , p<0.001 , Cohen’s d=−0.82 , 95% CI [−1.13,−0.52] . No significant differences were observed in the control group on the following dimensions: perspective-taking, F(1,36)=0.76 , p=0.386 , $\eta_{p}^{2}<0.01$ , 90% CI [0.00,0.05] ; empathic concern, F(1,36)=1.86 , p=0.175 , $\eta_{p}^{2}=0.02$ , 90% CI [0.00,0.07] .

The impact of the “group × time” interaction was significant on the empathic concern dimension, F(2,116)=3.48 , p=0.034 , $\eta_{p}^{2}=0.06$ , 90% CI [0.00,0.13] , but not significant for the perspective-taking dimension, F(2,116)=1.01 , p=0.368 , $\eta_{p}^{2}=0.02$ , 90% CI [0.00,0.06] .

#### 3.4.3. Self-compassion

In the case of self-compassion (Table 3), no significant differences were observed between the groups before the intervention ( F(2,116)=0.03 , p=0.969 , $\eta_{p}^{2}<0.01$ , 90% CI [0.00,0.00] ). In the d-MBI group, a significant main effect of “time” on self-compassion was observed, F(1,38)=4.29 , p=0.041 , $\eta_{p}^{2}=0.04$ , 90% CI [0.00,0.11] . A significant increase in self-compassion scores was observed from pre- to post-intervention, t(1,38)=−2.07 , p=0.041 , Cohen’s d=−0.33 , 95% CI [−0.65,−0.01] . In the f-MBI group, a main effect of “time” was also identified, F(1,42)=6.91 , p=0.010 , $\eta_{p}^{2}=0.06$ , 90% CI [0.01,0.14] . A significant increase in self-compassion scores was identified from pre- to post-intervention, t(1,42)=−2.63 , p=0.010 , Cohen’s d=−0.40 , 95% CI [−0.71,−0.10] . In the control group, no main effect of “time” was observed, F(1,36)=0.43 , p=0.512 , $\eta_{p}^{2}<0.01$ , 90% CI [0.00,0.04] . The “group × time” interaction was not significant, F(2,116)=0.91 , p=0.406 , $\eta_{p}^{2}=0.02$ , 90% CI [0.00,0.06] .

### 3.5. Influence of the MBIs on the online donations

A two-way ANOVA was conducted, with the number of donated grains as the dependent variable and gender (male and female) and group (d-MBI, f-MBI, and control) as independent variables. This revealed a significant main effect of the intervention, F(2,113)=18.97 , p<0.001 , $\eta_{p}^{2}=0.25$ , 90% CI [0.14,0.35] . Multiple comparisons (Bonferroni adjustment) demonstrated that participants in the d-MBI group ( M=509.74 , SD=197.08 ) and f-MBI group ( M=558.84 , SD=217.65 ) donated significantly more rice than participants in the control group ( M=300.81 , SD=145.67 ; males in d-MBI and control, t(1,113)=−3.27 , p=0.004 , Cohen’s d=−1.05 , 95% CI [−1.83,−0.27] ; females in d-MBI and control, t(1,113)=−3.36 , p=0.003 , Cohen’s d=−1.12 , 95% CI [−1.92,−0.31] ; males in f-MBI and control, t(1,113)=−3.90 , p<0.001 , Cohen’s d=−1.25 , 95% CI [−2.04,−0.47] ; females in f-MBI and control, t(1,113)=−4.39 , p<0.001 , Cohen’s d=−1.41 , 95% CI [−2.18,−0.63] ). There was no significant difference between the d-MBI and f-MBI groups (males, t(1,113)=−0.61 , p=1.00 , Cohen’s d=−0.20 , 95% CI [−1.01,0.60] ; females, t(1,113)=−0.97 , p=1.00 , Cohen’s d=−0.29 , 95% CI [−1.01,0.43] ). Gender had no significant main effect, F(1,113)=0.02 , p=0.893 , $\eta_{p}^{2}<0.01$ , 90% CI [0.00,0.01] and the interaction was also not significant, F(2,113)=0.06 , p=0.946 , $\eta_{p}^{2}<0.01$ , 90% CI [0.00,0.00] .

## 4. Discussion

The findings of this study provide support for Hypothesis 1, as it was found that participants who received mindfulness interventions spent more time donating rice to charitable organizations compared to participants in the active control group. Thus, this finding supports previous research (e.g., Donald et al., 2019; Schindler and Friese, 2022), suggesting that enhancing mindfulness promotes prosocial behavior. Specifically, increased mindfulness leads to higher empathic concern (Donald et al., 2019; Berry et al., 2020). Empathic concern (or compassion) can be defined as the response to the suffering of others (Pfattheicher et al., 2016). Mindfulness interventions typically help to disengage and decouple from mental content through non-judgmental acceptance (Schindler and Friese, 2022). Cultivating this ability weakens self-referential thoughts and emotions, further reduces the boundaries between self and others, and thus increases empathy for others in need (Berry et al., 2020). Our study confirms that this effect is present in interactions with real-world charitable organizations, where time spent is the requested donation. Furthermore, results confirm that, by using time as currency, mindfulness interventions can lead to more sustained prosocial behaviors to alleviate negative emotions, which is similar to the instantaneous effects of mindfulness practices found in previous research (e.g., O’Connor et al., 2022; Do et al., 2023).

This study found an interesting discrepancy: participants in the d-MBI group did not spend as much time donating rice to the WFP as predicted compared to participants in the f-MBI group (Hypothesis 2 was not supported). Why were the prosocial effects more pronounced in the f-MBI group? One possible explanation is the influence of familiarity between students and mindfulness instructors on the acceptability, engagement, and dropout rates of MBIs (González-García et al., 2021). Previous research emphasized the potential importance of student-teacher familiarity (Hill and Jones, 2018). Teachers familiar with their students often maintain a relatively optimistic about learning outcomes, thus prompting students’ higher achievement levels (Hill and Jones, 2021). Conversely, familiarity with their teachers tends to spur students to participate in classroom activities more enthusiastically and feel more secure (Hwang et al., 2021). On the other hand, the d-MBI group was introduced to the concept through mini-lectures, which only included video footage of mindfulness instructors from the waist up, explaining the main themes; all intervention audios were recorded in a different teacher’s voice. These factors may explain the more potent effects of the f-MBI group intervention. It can be seen that some of the differences in participation and outcomes of digital mental health interventions may be caused by common and related factors embedded in the technology itself (Cavanagh and Millings, 2013). Future research can assess the unique contributions of these related factors to the intervention results. Moreover, our findings align with Fischer et al.’s (2020) research, which suggests that while self-guided interventions accessible via smartphones and web-based applications are effective for mental health improvement, they do not perform as well as face-to-face and group-based therapeutic interventions. Hence, they should not replace clinical interventions for individuals and groups in need.

Our study results also extend a small but growing body of research that suggests mindfulness training can increase empathy and self-compassion. Compared with the active control group, the intervention group participants significantly improved their social connection, and reported high empathy and self-compassion pre-post scores, with medium effects for all evaluated variables. This is consistent with previous research on MBIs, including a recent meta-analysis investigating MBIs in children and adolescents (Cheang et al., 2019), a study demonstrating the mediating role of social connections between mindfulness and mental health (Rehman et al., 2021), and a study investigating increased salivary oxytocin and empathy after MBIs (Bellosta-Batalla et al., 2020a). All these studies evaluated randomized controlled trials. However, the results of this study were slightly lower than similar studies providing mindfulness interventions for adolescents (e.g., Hawk et al., 2013; Edwards et al., 2014; Bluth et al., 2016; Bluth and Eisenlohr-Moul, 2017; Yang and Kang, 2020). We attribute this discrepancy to the increased anxiety and stress experienced by adolescents during the COVID-19 pandemic lockdowns. This stringent confinement, alongside the threat of rising infection rates, led to significant psychological distress and severe mental health consequences (Brooks et al., 2020; González-Sanguino et al., 2020), especially in vulnerable groups such as minors (Hollenstein et al., 2021; Magson et al., 2021). Concurrently, the stress caused by the overconsumption of social media has deepened the profound psychological impact of the lockdown on adolescents (Dong et al., 2020; Marciano et al., 2022). Indeed, during the COVID-19 crisis, the spread of misinformation, fake news, and shocking images on social media potentially exacerbated feelings of fear, anxiety, stress, and worry (Zhao and Zhou, 2020; Lopes et al., 2022). Notably, self-compassion has been identified as a crucial correlate of stress coping. When individuals experience negative emotions or events, self-compassion may be a valuable coping resource, potentially preventing the occurrence of psychological distress (Ewert et al., 2021). Digital media resolve some methodological challenges associated with traditional face-to-face interventions, such as the feasibility of students independently conducting short-term mindfulness meditation training during the COVID-19 home isolation. Therefore, for policymakers, public health agencies, and educational institutions, introducing d-MBIs is a valuable strategy to promote adolescents’ mental health and provide them with effective resources for emotional regulation and coping with challenges.

An essential yet underexplored question concerns the required duration of mindfulness practice for emotional improvement. While some research suggests that just one 5-min meditation session can increase positive emotions (Ridderinkhof et al., 2017), other studies report that a significant stress reduction requires at least four weeks of continuous practice (Baer et al., 2012). In the current study, a single 90-min session of MBIs was sufficient to boost empathy and compassion, foster pro-social behavior, and enhance well-being. This aligns with a previous report that 30 min of MBIs enhanced students’ empathy and increased oxytocin levels in saliva (Bellosta-Batalla et al., 2020b). These findings may help lessen the economic and public health burdens associated with mental disorders like anxiety, stress, and depression (Wiegner et al., 2015), particularly given the affordability and accessibility of digital health platforms. Despite these promising results, substantial additional research is required before definitive conclusions can be reached.

In conclusion, this research contributes to assessing the efficacy of MBIs delivered to adolescents via technology—a medium that resonates with this age group, given their overall affinity for electronic devices and the internet. Digital platforms may potentially solve some methodological challenges associated with conventional interventions (such as mindfulness-based stress reduction). For instance, quantifying the amount or “dosage” of mindfulness training in meditation research often presents a challenge (Davidson and Kaszniak, 2015). This is particularly true for interventions involving self-reported home practice or active control, which cannot be directly measured. Hence, d-MBIs could be a valuable tool for primary and secondary prevention in maintaining mental health (González-García et al., 2021). Adolescents have long been seen as self-centered and rebellious. However, this study aims to clarify that d-MBIs are an effective method for promoting social and psychological health among adolescents, meeting their basic need to contribute to others and gain the capacity to consider others. This is a crucial step in researching how adolescents can incorporate mindfulness into their daily lives over the long term. Although this modality of MBI encounters various challenges, they can be surmounted by applying some best practices in digital learning (Mrazek et al., 2019a,b; González-García et al., 2021), as considered in the current study. By capitalizing on the benefits of digital platforms, we can optimize the delivery and impact of mindfulness-based interventions for adolescents, enhancing their mental health and overall well-being.

## 5. Limitations and future research

A series of limitations should be considered when evaluating the results of this study. First, the results are limited by a moderate sample size. Although we did not find baseline differences in sociodemographic variables among participants assigned to different groups, and we do not have any apparent reason to assume that the characteristics of participants under different conditions have other baseline differences (as explained in the Method section), we must interpret the causal relationship of the effects we reported cautiously. Future research should try to replicate and expand our results with larger research cohorts to address the current limitations.

Second, the current study did not thoroughly compare the differences in student experience between virtual instructor representations in d-MBIs and real instructor representations in f-MBIs. This area warrants further exploration, as such a comparison could provide insights into how to improve both online and in-person intervention methods. Although student-teacher familiarity is a factor in mindfulness interventions, the experiences may vary among students. d-MBIs offer less opportunity for personalized interaction, feedback, and the adjustment of teaching according to students’ specific needs, which could potentially lower the effectiveness of the intervention. Furthermore, instructor bias is a crucial factor affecting f-MBIs. If instructors have positive or negative preconceptions about certain students, it could influence their evaluation and teaching methods, thereby affecting the outcomes of the intervention. Future research should focus on developing more structured, standardized intervention measures to minimize individual differences in intervention results. Simultaneously, instructors need to learn how to adjust their teaching strategies within a guiding framework to meet the needs of each student. This may involve instructor training, the development of educational technology, and research on how positive thinking interventions can be applied in different environments. It would ensure the intervention measures’ consistency while catering to different students’ needs.

Third, the Freerice task is actually a test of knowledge, which may explain why some students stopped the game because they found it too tricky rather than losing the motivation to display pro-social behaviors. However, this is expected to have only a minimal impact, as all participants are high school students from key classes, so they are expected to have a sufficiently complex vocabulary to meet the requirements of this task.

Lastly, we only studied one type of charitable behavior. The advantage of the target behavior is that it is objectively and precisely measured, and in this case, the manipulation of the mindfulness intervention was feasible. Future research should determine whether our findings can be extended to other pro-social behaviors. Additionally, the behavioral cost of the tasks on www.freerice.com is relatively low, raising the question of whether these results would generalize to higher-cost behaviors. Engel’s (2011) meta-analysis suggests no or only minor effects of stake size on generosity.

## 6. Conclusion

MBIs can effectively improve empathy and compassion in children and adolescents, so there is a great need for accessible mental health promotion tools. These tools are increasingly provided digitally, with the field of digital mental health tools rapidly developing along with technological advances. This study of digital tool schemes and their effectiveness indicates that d-MBIs can enhance empathy and self-compassion in adolescents, promoting online charitable behavior. The results suggest that while the effect size of d-MBIs is less than that of traditional f-MBIs, they are more effective than control groups (e.g., Spijkerman et al., 2016; Stratton et al., 2017). This is a positive indication, bolstering the general recommendation for using these digital interventions, even in scenarios where immediate clinical guidance or supervision is not readily available. Nonetheless, the most beneficial approach for adolescents is a school-based environment, a degree of guidance and professional support, and consistent adherence to interventions. Our research demonstrates the value of d-MBIs in academic terms and how they can positively impact modern society’s charitable endeavors. Future research should pay particular attention to diversity-sensitive design and content and the ongoing availability of the tools developed.

## Data availability statement

The original contributions presented in the study are included in the article/supplementary material, further inquiries can be directed to the corresponding author.

## Ethics statement

The studies involving human participants were reviewed and approved by the Harbin Institute of Technology's Ethics Committee. Written informed consent to participate in this study was provided by the participants' legal guardian/next of kin. Before the experiment, all participants gave their written informed consent and were compensated for their involvement.

## Author contributions

MH, DL, and TL proposed the research topic and designed the research framework. MH, TL, and SL collected and analyzed the data and drew tables and figures. MH and TL drafted the manuscript and made several important revisions. All the authors approved the submission of the final version.

## Conflict of interest

The authors declare that the research was conducted in the absence of any commercial or financial relationships that could be construed as a potential conflict of interest.

## Publisher’s note

All claims expressed in this article are solely those of the authors and do not necessarily represent those of their affiliated organizations, or those of the publisher, the editors and the reviewers. Any product that may be evaluated in this article, or claim that may be made by its manufacturer, is not guaranteed or endorsed by the publisher.

## Footnotes

^1^www.freerice.com

^2^www.sojump.com

## References

[ref1] Antonson C. Thorsén F. Sundquist J. Sundquist K. (2018). Upper secondary school students’ compliance with two internet-based self-help programmes: a randomised controlled trial. Eur. Child Adolesc. Psychiatry 27, 191–200. doi: 10.1007/s00787-017-1035-6 28776094 PMC5842245

[ref2] Armstrong J. S. Overton T. S. (1977). Estimating nonresponse bias in mail surveys. J. Mark. Res. 14, 396–402. doi: 10.1177/002224377701400320

[ref3] Baer R. A. Carmody J. Hunsinger M. (2012). Weekly change in mindfulness and perceived stress in a mindfulness-based stress reduction program. J. Clin. Psychol. 68, 755–765. doi: 10.1002/jclp.21865, PMID: 22623334

[ref4] Batson C. D. (2010). Altruism in Humans. Oxford: Oxford University Press.

[ref5] Bellosta-Batalla M. Blanco-Gandía M. C. Rodríguez-Arias M. Cebolla A. Pérez-Blasco J. Moya-Albiol L. (2020a). Increased salivary oxytocin and empathy in students of clinical and Health Psychology after a mindfulness and compassion-based intervention. Mindfulness 11, 1006–1017. doi: 10.1007/s12671-020-01316-7

[ref6] Bellosta-Batalla M. del Carmen Blanco-Gandía M. Rodríguez-Arias M. Cebolla A. Pérez-Blasco J. Moya-Albiol L. (2020b). Brief mindfulness session improves mood and increases salivary oxytocin in psychology students. Stress. Health 36, 469–477. doi: 10.1002/smi.2942 32227624

[ref7] Bergin A. D. Vallejos E. P. Davies E. B. Daley D. Ford T. Harold G.. (2020). Preventive digital mental health interventions for children and young people: a review of the design and reporting of research. NPJ Digital Medicine 3:133. doi: 10.1038/s41746-020-00339-7 33083568 PMC7562906

[ref8] Berry D. R. Hoerr J. P. Cesko S. Alayoubi A. Carpio K. Zirzow H.. (2020). Does mindfulness training without explicit ethics-based instruction promote prosocial Behaviors? A meta-analysis. Personal. Soc. Psychol. Bull. 46, 1247–1269. doi: 10.1177/0146167219900418 31971095

[ref9] Bluth K. Eisenlohr-Moul T. A. (2017). Response to a mindful self-compassion intervention in teens: a within-person association of mindfulness, self-compassion, and emotional well-being outcomes. J. Adolesc. 57, 108–118. doi: 10.1016/j.adolescence.2017.04.001 28414965 PMC5514374

[ref10] Bluth K. Gaylord S. A. Campo R. A. Mullarkey M. C. Hobbs L. (2016). Making friends with yourself: a mixed methods pilot study of a mindful self-compassion program for adolescents. Mindfulness 7, 479–492. doi: 10.1007/s12671-015-0476-6 27110301 PMC4838201

[ref11] Brooker H. Wesnes K. A. Ballard C. Hampshire A. Aarsland D. Khan Z.. (2019). An online investigation of the relationship between the frequency of word puzzle use and cognitive function in a large sample of older adults. Int. J. Geriatr. Psychiatry 34, 921–931. doi: 10.1002/gps.5033 30443984

[ref12] Brooks S. K. Webster R. K. Smith L. E. Woodland L. Wessely S. Greenberg N.. (2020). The psychological impact of quarantine and how to reduce it: rapid review of the evidence. Lancet 395, 912–920. doi: 10.1016/S0140-6736(20)30460-8 32112714 PMC7158942

[ref13] Cameron C. D. Fredrickson B. L. (2015). Mindfulness facets predict helping behavior and distinct helping-related emotions. Mindfulness 6, 1211–1218. doi: 10.1007/s12671-014-0383-2

[ref14] Cavanagh K. Millings A. (2013). (inter)personal computing: the role of the therapeutic relationship in E-mental health. J. Contemp. Psychother. 43, 197–206. doi: 10.1007/s10879-013-9242-z

[ref15] Cheang R. Gillions A. Sparkes E. (2019). Do mindfulness-based interventions increase empathy and compassion in children and adolescents: a systematic review. J. Child Fam. Stud. 28, 1765–1779. doi: 10.1007/s10826-019-01413-9

[ref16] Davidson R. J. Kaszniak A. W. (2015). Conceptual and methodological issues in research on mindfulness and meditation. Am. Psychol. 70, 581–592. doi: 10.1037/a0039512 26436310 PMC4627495

[ref17] Denham S. A. McKinley M. Couchoud E. A. Holt R. (1990). Emotional and Behavioral predictors of preschool peer ratings. Child Dev. 61, 1145–1152. doi: 10.1111/j.1467-8624.1990.tb02848.x 2209184

[ref18] Do H. Hoang H. Nguyen N. An A. Chau H. Khuu Q.. (2023). Intermediate effects of mindfulness practice on the brain activity of college students: An EEG study. IBRO Neurosci. Rep. 14, 308–319. doi: 10.1016/j.ibneur.2023.03.003 37388488 PMC10300454

[ref19] Donald J. N. Sahdra B. K. Van Zanden B. Duineveld J. J. Atkins P. W. B. Marshall S. L.. (2019). Does your mindfulness benefit others? A systematic review and meta-analysis of the link between mindfulness and prosocial behaviour. Br. J. Psychol. 110, 101–125. doi: 10.1111/bjop.12338 30094812

[ref20] Dong H. Yang F. Lu X. Hao W. (2020). Internet addiction and related psychological factors among children and adolescents in China during the coronavirus disease 2019 (COVID-19) epidemic. Front. Psych. 11:751. doi: 10.3389/fpsyt.2020.00751 PMC7492537 32982806

[ref21] Du Y. Wei M. (2015). Acculturation, enculturation, social connectedness, and subjective well-being among Chinese international students. Couns. Psychol. 43, 299–325. doi: 10.1177/0011000014565712

[ref22] Edwards M. Adams E. M. Waldo M. Hadfield O. D. Biegel G. M. (2014). Effects of a mindfulness group on Latino adolescent students: examining levels of perceived stress, mindfulness, self-compassion, and psychological symptoms. J. Special. Group Work 39, 145–163. doi: 10.1080/01933922.2014.891683

[ref23] Eisenberg N. Miller P. A. (1987). The relation of empathy to prosocial and related behaviors. Psychol. Bull. 101, 91–119. doi: 10.1037/0033-2909.101.1.91 3562705

[ref24] Engel C. (2011). Dictator games: a meta study. Exp. Econ. 14, 583–610. doi: 10.1007/s10683-011-9283-7

[ref25] Ewert C. Vater A. Schröder-Abé M. (2021). Self-compassion and coping: a meta-analysis. Mindfulness 12, 1063–1077. doi: 10.1007/s12671-020-01563-8

[ref26] Farrelly D. Bennett M. (2018). Empathy leads to increased online charitable behaviour when time is the currency. J. Community Appl. Soc. Psychol. 28, 42–46. doi: 10.1002/casp.2339

[ref27] Ferrari M. Beath A. Einstein D. A. Yap K. Hunt C. (2022). Gender differences in self-compassion: a latent profile analysis of compassionate and uncompassionate self-relating in a large adolescent sample. Curr. Psychol. doi: 10.1007/s12144-022-03408-0

[ref28] Fischer R. Bortolini T. Karl J. A. Zilberberg M. Robinson K. Rabelo A.. (2020). Rapid review and meta-meta-analysis of self-guided interventions to address anxiety, depression, and stress during COVID-19 social distancing. Front. Psychol. 11:3876. doi: 10.3389/fpsyg.2020.563876 PMC7655981 33192837

[ref29] González-García M. Álvarez J. C. Pérez E. Z. Fernandez-Carriba S. López J. G. (2021). Feasibility of a brief online mindfulness and compassion-based intervention to promote mental health among university students during the COVID-19 pandemic. Mindfulness 12, 1685–1695. doi: 10.1007/s12671-021-01632-6 34025814 PMC8127469

[ref30] González-Sanguino C. Ausín B. Castellanos M. Á. Saiz J. López-Gómez A. Ugidos C.. (2020). Mental health consequences during the initial stage of the 2020 coronavirus pandemic (COVID-19) in Spain. Brain Behav. Immun. 87, 172–176. doi: 10.1016/j.bbi.2020.05.040 32405150 PMC7219372

[ref31] Graber J. A. Brooks-Gunn J. (1996). Transitions and turning points: navigating the passage from childhood through adolescence. Dev. Psychol. 32, 768–776. doi: 10.1037/0012-1649.32.4.768

[ref32] Grepmair L. Mitterlehner F. Loew T. Bachler E. Rother W. Nickel M. (2007). Promoting mindfulness in psychotherapists in training influences the treatment results of their patients: a randomized, double-blind, controlled study. Psychother. Psychosom. 76, 332–338. doi: 10.1159/000107560 17917468

[ref33] Hall-Lande J. A. Eisenberg M. E. Christenson S. L. Neumark-Sztainer D. (2007). Social isolation, psychological health, and protective factors in adolescence. Adolescence 42, 265–286. 17849936

[ref34] Hawk S. T. Keijsers L. Branje S. J. T. Graaff J. V. de Wied M. Meeus W. (2013). Examining the interpersonal reactivity index (IRI) among early and late adolescents and their mothers. J. Pers. Assess. 95, 96–106. doi: 10.1080/00223891.2012.696080 22731809

[ref35] Hill A. J. Jones D. B. (2018). A teacher who knows me: the academic benefits of repeat student-teacher matches. Econ. Educ. Rev. 64, 1–12. doi: 10.1016/j.econedurev.2018.03.004

[ref36] Hill A. J. Jones D. B. (2021). Self-fulfilling prophecies in the classroom. J. Hum. Cap. 15, 400–431. doi: 10.1086/715204

[ref37] Hoge E. A. Acabchuk R. L. Kimmel H. Moitra E. Britton W. B. Dumais T.. (2021). Emotion-related constructs engaged by mindfulness-based interventions: a systematic review and meta-analysis. Mindfulness 12, 1041–1062. doi: 10.1007/s12671-020-01561-w 34149957 PMC8210838

[ref38] Hollenstein T. Colasante T. Lougheed J. P. (2021). Adolescent and maternal anxiety symptoms decreased but depressive symptoms increased before to during COVID-19 lockdown. J. Res. Adolesc. 31, 517–530. doi: 10.1111/jora.12663 34448298 PMC8646576

[ref39] Hong J. M. Lee W.-N. (2021). The stages-of-change approach for prosocial behavior: message tailoring to encourage blood donation. J. Appl. Soc. Psychol. 51, 219–236. doi: 10.1111/jasp.12727

[ref40] Howells A. Ivtzan I. Eiroa-Orosa F. J. (2016). Putting the ‘app’ in happiness: a randomised controlled trial of a smartphone-based mindfulness intervention to enhance wellbeing. J. Happiness Stud. 17, 163–185. doi: 10.1007/s10902-014-9589-1

[ref41] Huang X. Li W. Sun B. Chen H. Davis M. H. (2012). The validation of the interpersonal reactivity index for Chinese teachers from primary and middle schools. J. Psychoeduc. Assess. 30, 194–204. doi: 10.1177/0734282911410588

[ref42] Hwang N. Kisida B. Koedel C. (2021). A familiar face: student-teacher rematches and student achievement. Econ. Educ. Rev. 85:102194. doi: 10.1016/j.econedurev.2021.102194

[ref43] Kabat-Zinn J. (2015). Mindfulness. Mindfulness 6, 1481–1483. doi: 10.1007/s12671-015-0456-x

[ref44] Kieling C. Baker-Henningham H. Belfer M. Conti G. Ertem I. Omigbodun O.. (2011). Child and adolescent mental health worldwide: evidence for action. Lancet (London, England) 378, 1515–1525. doi: 10.1016/s0140-6736(11)60827-1 22008427

[ref46] Kock N. (2015). Common method bias in PLS-SEM: a full collinearity assessment approach. Int. J. e-Collab. 11, 1–10. doi: 10.4018/ijec.2015100101

[ref47] Kreplin U. Farias M. Brazil I. A. (2018). The limited prosocial effects of meditation: a systematic review and meta-analysis. Sci. Rep. 8:2403. doi: 10.1038/s41598-018-20299-z 29402955 PMC5799363

[ref48] La Greca A. M. Harrison H. M. (2005). Adolescent peer relations, friendships, and romantic relationships: Do they predict social anxiety and depression? J. Clin. Child Adolesc. Psychol. 34, 49–61. doi: 10.1207/s15374424jccp3401_5 15677280

[ref49] Lee R. M. Robbins S. B. (1995). Measuring belongingness: the social connectedness and the social assurance scales. J. Couns. Psychol. 42, 232–241. doi: 10.1037/0022-0167.42.2.232

[ref50] Lemay V. Hoolahan J. Buchanan A. (2019). Impact of a yoga and meditation intervention on students’ stress and anxiety levels. Am. J. Pharm. Educ. 83:7001. doi: 10.5688/ajpe7001 31333265 PMC6630857

[ref51] Lin J. W. Mai L. J. (2018). Impact of mindfulness meditation intervention on academic performance. Innov. Educ. Teach. Int. 55, 366–375. doi: 10.1080/14703297.2016.1231617

[ref52] Lockwood P. L. Hamonet M. Zhang S. H. Ratnavel A. Salmony F. U. Husain M.. (2017). Prosocial apathy for helping others when effort is required. Nat. Hum. Behav. 1:0131. doi: 10.1038/s41562-017-0131 28819649 PMC5555390

[ref53] Lopes L. S. Valentini J. P. Monteiro T. H. Costacurta M. C. F. Soares L. O. N. Telfar-Barnard L.. (2022). Problematic social media use and its relationship with depression or anxiety: a systematic review. Cyberpsychol. Behav. Soc. Netw. 25, 691–702. doi: 10.1089/cyber.2021.0300 36219756

[ref54] Luberto C. M. Shinday N. Song R. Philpotts L. L. Park E. R. Fricchione G. L.. (2018). A systematic review and meta-analysis of the effects of meditation on empathy, compassion, and prosocial Behaviors. Mindfulness 9, 708–724. doi: 10.1007/s12671-017-0841-8 30100929 PMC6081743

[ref55] Lucas-Thompson R. G. Broderick P. C. Coatsworth J. D. Smyth J. M. (2019). New avenues for promoting mindfulness in adolescence using mHealth. J. Child Fam. Stud. 28, 131–139. doi: 10.1007/s10826-018-1256-4 31160875 PMC6544044

[ref56] Ly K. H. Asplund K. Andersson G. (2014). Stress management for middle managers via an acceptance and commitment-based smartphone application: a randomized controlled trial. Internet Interv. 1, 95–101. doi: 10.1016/j.invent.2014.06.003

[ref57] Macchia L. Farmer J. Kubzansky L. D. (2023). Prosocial behaviour helps to ease physical pain: longitudinal evidence from Britain. J. Psychosom. Res. 169:111325. doi: 10.1016/j.jpsychores.2023.111325 37037156

[ref58] Magson N. R. Freeman J. Y. A. Rapee R. M. Richardson C. E. Oar E. L. Fardouly J. (2021). Risk and protective factors for prospective changes in adolescent mental health during the COVID-19 pandemic. J. Youth Adolesc. 50, 44–57. doi: 10.1007/s10964-020-01332-9 33108542 PMC7590912

[ref59] Mani M. Kavanagh D. J. Hides L. Stoyanov S. R. (2015). Review and evaluation of mindfulness-based iPhone Apps. JMIR Mhealth Uhealth 3:e82. doi: 10.2196/mhealth.4328 26290327 PMC4705029

[ref60] Marciano L. Ostroumova M. Schulz P. J. Camerini A.-L. (2022). Digital media use and adolescents’ mental health during the COVID-19 pandemic: a systematic review and meta-analysis. Front. Public Health 9:3868. doi: 10.3389/fpubh.2021.793868 PMC8848548 35186872

[ref61] Marshall S. L. Ciarrochi J. Parker P. D. Sahdra B. K. (2020). Is self-compassion selfish? The development of self-compassion, empathy, and prosocial behavior in adolescence. J. Res. Adolesc. 30, 472–484. doi: 10.1111/jora.12492 30884003

[ref62] Mason M. C. Zamparo G. Marini A. Ameen N. (2022). Glued to your phone? Generation Z’s smartphone addiction and online compulsive buying. Comput. Hum. Behav. 136:107404. doi: 10.1016/j.chb.2022.107404

[ref63] Matland R. E. Murray G. R. (2016). I only have eyes for you: does implicit social pressure increase voter turnout? Polit. Psychol. 37, 533–550. doi: 10.1111/pops.12275

[ref64] Meiklejohn J. Phillips C. Freedman M. L. Griffin M. L. Biegel G. Roach A.. (2012). Integrating mindfulness training into K-12 education: fostering the resilience of teachers and students. Mindfulness 3, 291–307. doi: 10.1007/s12671-012-0094-5

[ref65] Mrazek A. J. Mrazek M. D. Cherolini C. M. Cloughesy J. N. Cynman D. J. Gougis L. J.. (2019a). The future of mindfulness training is digital, and the future is now. Curr. Opin. Psychol. 28, 81–86. doi: 10.1016/j.copsyc.2018.11.012 30529975

[ref66] Mrazek A. J. Mrazek M. D. Reese J. V. Kirk A. C. Gougis L. J. Delegard A. M.. (2019b). Mindfulness-based attention training: feasibility and preliminary outcomes of a digital course for high school students. Educ. Sci. 9:230. doi: 10.3390/educsci9030230

[ref04] Mu W. Chen Z. Duan W. (2021). An Extended Evaluation of Academic Encouragement Scale for Adolescents. J. Psychoeduc. Assess. 39, 332–345. doi: 10.1177/0734282920977723

[ref67] Norris C. J. Creem D. Hendler R. Kober H. (2018). Corrigendum: brief mindfulness meditation improves attention in novices: evidence from ERPs and moderation by neuroticism. Front. Hum. Neurosci. 12:342. doi: 10.3389/fnhum.2018.00342 30220906 PMC6134230

[ref68] O’Connor E. J. Murphy A. Kohler M. J. Chan R. W. Immink M. A. (2022). Instantaneous effects of mindfulness meditation on tennis return performance in elite junior athletes completing an implicitly sequenced serve return task. Front. Sports Active Living 4:7654. doi: 10.3389/fspor.2022.907654 PMC9446240 36081619

[ref69] Pfattheicher S. Sassenrath C. Schindler S. (2016). Feelings for the suffering of others and the environment: compassion fosters Proenvironmental tendencies. Environ. Behav. 48, 929–945. doi: 10.1177/0013916515574549

[ref70] Plaza García I. Sánchez C. M. Espílez Á. S. García-Magariño I. Guillén G. A. García-Campayo J. (2017). Development and initial evaluation of a mobile application to help with mindfulness training and practice. Int. J. Med. Inform. 105, 59–67. doi: 10.1016/j.ijmedinf.2017.05.018 28750912

[ref01] Podsakoff P. M. MacKenzie S. B. Lee J.-Y. Podsakoff N. P. (2003). Common method biases in behavioral research: A critical review of the literature and recommended remedies. J. Appl. Psychol. 88, 879–903. doi: 10.1037/0021-9010.88.5.879 14516251

[ref02] Podsakoff P. M. Organ D. W. (1986). Self-reports in organizational research: problems and prospects. J. Manag. 12, 531–544. doi: 10.1177/014920638601200408

[ref71] Raes F. Pommier E. Neff K. D. Van Gucht D. (2011). Construction and factorial validation of a short form of the self-compassion scale. Clin. Psychol. Psychother. 18, 250–255. doi: 10.1002/cpp.702 21584907

[ref03] Rehman A. U. You X. Wang Z. Kong F. (2021). The link between mindfulness and psychological well-being among university students: The mediating role of social connectedness and self-esteem. Curr. Psychol. 42, 11772–11781. doi: 10.1007/s12144-021-02428-6

[ref72] Ridderinkhof A. de Bruin E. I. Brummelman E. Bögels S. M. (2017). Does mindfulness meditation increase empathy? An experiment. Self Identity 16, 251–269. doi: 10.1080/15298868.2016.1269667

[ref73] Schindler S. Friese M. (2022). The relation of mindfulness and prosocial behavior: what do we (not) know? Curr. Opin. Psychol. 44, 151–156. doi: 10.1016/j.copsyc.2021.09.010 34662774

[ref74] Schram A. (2005). Artificiality: the tension between internal and external validity in economic experiments. J. Econ. Methodol. 12, 225–237. doi: 10.1080/13501780500086081

[ref75] Semple R. J. Droutman V. Reid B. A. (2017). Mindfulness goes to school: things learned (so far) from research and real-world experiences. Psychol. Sch. 54, 29–52. doi: 10.1002/pits.21981 28458403 PMC5405439

[ref76] Sommers-Spijkerman M. Austin J. Bohlmeijer E. Pots W. (2021). New evidence in the booming field of online mindfulness: An updated meta-analysis of randomized controlled trials. JMIR Ment Health 8:e28168. doi: 10.2196/28168 34279240 PMC8329762

[ref77] Spijkerman M. P. J. Pots W. T. M. Bohlmeijer E. T. (2016). Effectiveness of online mindfulness-based interventions in improving mental health: a review and meta-analysis of randomised controlled trials. Clin. Psychol. Rev. 45, 102–114. doi: 10.1016/j.cpr.2016.03.009 27111302

[ref78] Steiger J. H. (2004). Beyond the F test: effect size confidence intervals and tests of close fit in the analysis of variance and contrast analysis. Psychol. Methods 9, 164–182. doi: 10.1037/1082-989X.9.2.164 15137887

[ref79] Stratton E. Lampit A. Choi I. Calvo R. A. Harvey S. B. Glozier N. (2017). Effectiveness of eHealth interventions for reducing mental health conditions in employees: a systematic review and meta-analysis. PLoS One 12:e0189904. doi: 10.1371/journal.pone.0189904 29267334 PMC5739441

[ref80] Telzer E. H. Fuligni A. J. Lieberman M. D. Miernicki M. E. Galván A. (2014). The quality of adolescents’ peer relationships modulates neural sensitivity to risk taking. Soc. Cogn. Affect. Neurosci. 10, 389–398. doi: 10.1093/scan/nsu064 24795443 PMC4350483

[ref81] van Emmerik A. A. P. Berings F. Lancee J. (2018). Efficacy of a mindfulness-based Mobile application: a randomized waiting-list controlled trial. Mindfulness 9, 187–198. doi: 10.1007/s12671-017-0761-7 29387266 PMC5770479

[ref82] Wahbeh H. Svalina M. N. Oken B. S. (2014). Group, one-on-one, or internet? Preferences for mindfulness meditation delivery format and their predictors. Open Med. J. 1, 66–74. doi: 10.2174/1874220301401010066 27057260 PMC4820831

[ref83] Wallmark E. Safarzadeh K. Daukantaitė D. Maddux R. E. (2013). Promoting altruism through meditation: An 8-week randomized controlled pilot study. Mindfulness 4, 223–234. doi: 10.1007/s12671-012-0115-4

[ref84] Wiegner L. Hange D. Björkelund C. Ahlborg G. (2015). Prevalence of perceived stress and associations to symptoms of exhaustion, depression and anxiety in a working age population seeking primary care – an observational study. BMC Fam. Pract. 16:38. doi: 10.1186/s12875-015-0252-7 25880219 PMC4377029

[ref85] Wu R. Liu L.-L. Zhu H. Su W.-J. Cao Z.-Y. Zhong S.-Y.. (2019). Brief mindfulness meditation improves emotion processing. Front. Neurosci. 13:1074. doi: 10.3389/fnins.2019.01074 31649501 PMC6795685

[ref86] Yang H. Kang S.-J. (2020). Exploring the Korean adolescent empathy using the interpersonal reactivity index (IRI). Asia Pac. Educ. Rev. 21, 339–349. doi: 10.1007/s12564-019-09621-0

[ref87] Zhao N. Zhou G. (2020). Social media use and mental health during the COVID-19 pandemic: moderator role of disaster stressor and mediator role of negative affect. Appl. Psychol. Health Well Being 12, 1019–1038. doi: 10.1111/aphw.12226 32945123 PMC7536964

[ref88] Zhu S. Xu Z. Dong Y. Xiong N. Wang Y. (2022). What will the future kitchen look like? An exploratory laboratory study of the future expectations of Chinese generation Z. Int. J. Ind. Ergon. 87:103259. doi: 10.1016/j.ergon.2021.103259

